# What are we measuring? A critique of range of motion methods currently in use for Dupuytren’s disease and recommendations for practice

**DOI:** 10.1186/s12891-016-0884-3

**Published:** 2016-01-13

**Authors:** Anna L. Pratt, Catherine Ball

**Affiliations:** Division of Occupational Therapy and Community Nursing, College of Health and Life Sciences, Brunel University, Kingston Lane, Uxbridge, UB8 3PH UK; Kennedy Institute, University of Oxford, Roosevelt Drive, Oxford, OX3 7FY UK

**Keywords:** Dupuytren’s disease, Range of motion, Assessment, Outcome measure, Recommendations

## Abstract

**Background:**

Range of motion is the most frequently reported measure used in practice to evaluate outcomes. A goniometer is the most reliable tool to assess range of motion yet, the lack of consistency in reporting prevents comparison between studies. The aim of this study is to identify how range of motion is currently assessed and reported in Dupuytren’s disease literature. Following analysis recommendations for practice will be made to enable consistency in future studies for comparability. This paper highlights the variation in range of motion reporting in Dupuytren’s disease.

**Methods:**

A Participants, Intervention, Comparison, Outcomes and Study design format was used for the search strategy and search terms. Surgery, needle fasciotomy or collagenase injection for primary or recurrent Dupuytren’s disease in adults were included if outcomes were monitored using range of motion to record change. A literature search was performed in May 2013 using subject heading and free-text terms to also capture electronic publications ahead of print. In total 638 publications were identified and following screening 90 articles met the inclusion criteria. Data was extracted and entered onto a spreadsheet for analysis. A thematic analysis was carried out to establish any duplication, resulting in the final range of motion measures identified.

**Results:**

Range of motion measurement lacked clarity, with goniometry reportedly used in only 43 of the 90 studies, 16 stated the use of a range of motion protocol. A total of 24 different descriptors were identified describing range of motion in the 90 studies. While some studies reported active range of motion, others reported passive or were unclear. Eight of the 24 categories were identified through thematic analysis as possibly describing the same measure, ‘lack of joint extension’ and accounted for the most frequently used.

**Conclusions:**

Published studies lacked clarity in reporting range of motion, preventing data comparison and meta-analysis. Percentage change lacks context and without access to raw data, does not allow direct comparison of baseline characteristics. A clear description of what is being measured within each study was required. It is recommended that range of motion measuring and reporting for Dupuytren’s disease requires consistency to address issues that fall into 3 main categories:-

- Definition of terms
- Protocol statement
- Outcome reporting

**Electronic supplementary material:**

The online version of this article (doi:10.1186/s12891-016-0884-3) contains supplementary material, which is available to authorized users.

## Background

Change in hand function should be assessed using a combination of physical measures and questionnaires [1]. The lack of extension of the hand is the reason why patients with Dupuytren’s disease often seek advice [2] and therefore a measurement that captures this should be included in evaluation of Dupuytren’s disease. Range of motion (ROM) has customarily been used to evaluate hand function [3, 4] and has been defined as the most commonly used physical outcome measure in Dupuytren’s disease literature [5]. Using a goniometer to assess ROM is regarded as an objective and reliable measure [6], therefore an obvious assessment tool for Dupuytren’s disease.

Bindra [7] highlights the importance of developing and adhering to protocols for assessments such as goniometry for reliable measurement to be taken. However, it has recently been reported that there is a continued lack of consistency with the variation in measures used in ROM reporting precluding comparison between studies [5] and thus warrants further analysis. Post et al [8] recently acknowledged the paucity of high level evidence in surgical literature and recommended the development of higher methodological and reporting standards to assist this issue. Giladi and Chung [3] have also reported concerns in deficiencies associated with outcomes measurement with lack of transparency and incomplete reporting of data also raised as a concern [9].

This discussion paper aims to identify and review the diversity of assessment and reporting of ROM in Dupuytren’s disease literature using systematic processes following PRISMA guidelines [10] (Additional file 1). The aim was not to evaluate the quality of the interventions used. Following analysis of the data a minimum set of ROM measurements are identified and recommendations for future practice made.

Range of motion is the most frequently reported measure used in practice to evaluate outcomes. A goniometer is the most reliable tool to assess range of motion yet, the lack of consistency in reporting prevents comparison between studies. The aim of this study is to identify how ROM is currently assessed and reported in Dupuytren’s disease literature. Following analysis recommendations for practice will be made to enable consistency in future studies for comparability. This paper highlights the variation in range of motion reporting in Dupuytren’s disease.

## Methods

A Participants, Intervention, Comparison, Outcomes and Study design (PICOS) format was used for the search strategy and search terms (Table 1). Publications were suitable for inclusion if published in the English language within the last 20 years.

**Table 1:** Search strategy and Search terms using PICOS analysis

	Definition	Main Search Terms for Ovid Medline Strategy (Subject heading (/) and free text terms ($ indicates truncation; adj3 finds terms within 2 words of each other)
Participants	Persons with Dupuytren’s disease	Dupuytren’s contracture/or dupuytren $
Intervention	Surgical treatment including percutaneous or collagenase injection for Dupuytren's disease of the hand.	Exp surgical procedures, operative/or surg$ or fasciectom$ or fasciotom$ or dermofasciectom$ or open palm or mccash or aponeurectom$ or aponeurotom$.mp. Injections/or injections, Intralesional/or inject$ or collagenases/ or microbial collagenases/or collagenase$
Comparisons	Not applicable	
Outcomes	Range of motion/movement	Exp treatment outcome/or outcome assessment (healthcare)/or outcome$ or hand$ adj3 function$/or exp range of motion, articular/or range of motion or follow-up studies/or disability evaluation/or disab$ adj3 eval$ or disab adj3 assess$ or efficac$
Study design	All Included:-Screen search results manually to include RCT’s and non-randomised controlled clinical trials, prospective and retrospective case series. Excluded:-Case studies and conference papers.	Screened using study eligibility tool

**Fig. 1:**
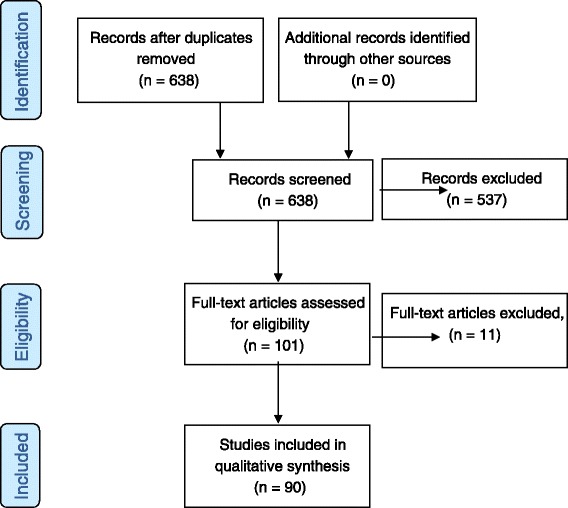
Study Search and Selection process, PRISMA flow diagram

A literature search was performed in May 2013 using subject heading and free-text terms as reported in Table 1. Ovid Medline, Embase and CINAHL (via EBSCOHost) databases were searched from 1992 to May 2013. In order to capture electronic publications ahead of print and not included in Ovid Medline, PubMed was searched from 2011 to May 2013. All searches were limited to publications in English and results were imported into in Refworks resulting in 638 publications after removal of duplicates (Fig. 1).

Using the review protocol, publications for this critique were suitable for inclusion if participants had received a surgical treatment, percutaneous fasciotomy or collagenase injection for Dupuytren’s disease, published in the English language and reported ROM outcomes that permitted analysis of the data. The follow-up period for post-intervention monitoring for included publications was not limited. Case studies and conference papers were excluded. All 638 titles/abstracts were independently screened by both authors using inclusion and exclusion criteria. If agreement was not initially reached both authors discussed the study to achieve a consensus of opinion. Full text was obtained for 101 publications that met the inclusion criteria. Using the study eligibility screening tool 11 publications were excluded as data did not enable analysis. In total 90 articles met the inclusion criteria and data extracted independently using the ROM reporting extraction form.

A Microsoft Excel (Microsoft, Seattle) spreadsheet was used to collect and tabulate data on intervention, population and ROM measure/s for each included study. The spreadsheet collected data on study design, reported ROM measure and if the method of measurement was described, identification if active or passive motion was recorded, if measurements were taken using a goniometer, whether a goniometry protocol was used, any classification or grading system reported and if hyperextension was reported how it was addressed in calculations.

The descriptions of the included articles ROM outcome variables were recorded verbatim for categories to be identified. A thematic analysis was carried out on the categories to establish any duplication, resulting in the final ROM measures identified.

## Results

### Study design

From the 90 included studies [11–100] 13 (14.4 %) were randomised controlled trials. The remainder of the studies were case series or cohort studies. An additional 26 (28.9 %) reported results from prospective studies and 31 (34.4 %) reported retrospectively. Two studies (2.2 %) used a combined prospective and retrospective design while the remaining 18 studies (20.0 %) were categorised as ‘unclear’ as it was not possible to ascertain if the design was prospective or retrospective. As the aim was to identify ROM measures used rather than to compare results of treatment further analysis of the overall quality of individual studies was not performed. The ROM reporting measure was synthesised at outcome level, as reported below.

### Goniometer and protocol use

Goniometry was explicitly used to measure ROM in 43 of the 90 studies and it was unclear how ROM was assessed in the remaining 47 studies, although results were reported in degrees. Of the 43 studies that reported use of a goniometry 16 stipulated a ROM protocol [12, 21, 23, 32, 38, 44, 48, 51, 53, 54, 63, 64, 68, 81, 99, 100]. It was unclear if a ROM protocol was used for the remaining 27 (62.8 %) studies that reported using a goniometer. In total 128 ROM data sets were reported from the 90 studies (Table 2) and in over 60 % of these it was unclear if measurements had been taken actively or passively. Of the 16 studies that reported using a goniometer protocol only 4 studies reported information about the measurement assessment position [32, 64, 68, 81]. Two studies [32, 81] reported placing the metacarpophalangeal (MCP) joint in flexion to assess passive proximal interphalangeal (PIP) joint extension and 2 studies [64, 81] assessed active PIP extension with the MCP flexed. A further study [68] stated the position of the wrist and hand.

**Table 2:** Distribution of papers reporting one or more ROM measure

		Frequency (n=)
1 ROM measure reported	[11–16, 18–21, 25–27, 29, 31–39, 45–49, 52, 55–57, 60–64, 70–74, 76, 79–88, 90–95, 97, 100]	61
2 ROM measures reported	[17, 22, 23, 40, 43, 51, 53, 54, 58, 59, 65–69, 75, 77, 78, 98, 99]	20
3 ROM measures reported	[24, 28, 30, 41, 42, 44, 50, 89, 96]	9

### Reported ROM measures

Nine studies used the maximum number of 3 ROM measures to report their results [24, 28, 30, 41, 42, 44, 50, 89, 96] Sixty one studies used 1 ROM measure and 20 used 2 ROM measures (Table 2).

Of the 61 studies reporting only one measure 9 studies [18, 25, 52, 62, 81, 86, 90–92] recorded passive motion only. Additionally two studies [48, 64] who used one measure reported both active and passive motion.

### Terminology

**Fig. 2:**
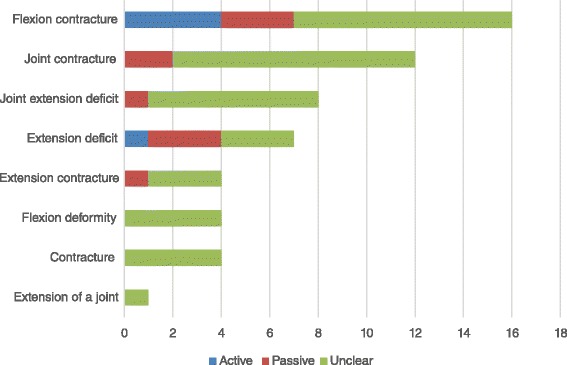
The identified 8 categories demonstrating the varied terminology for ‘lack of joint extension’ and frequency

The thematic analysis identified 24 ROM outcome categories. Further scrutiny revealed that 8 of the 24 described the same measure and for the purpose of this study were combined as a category describing ‘lack of extension’ (Fig. 2) resulting in a total of 17 categories. No study reported the use of more than one of these eight ‘Lack of joint extension’ terms. This supports the suggestion that the 8 categories describe the same measure. The resulting category ‘Lack of joint extension’ was used by 34 of 61 studies reporting one ROM measure, 12 of the 20 studies using 2 measures and all 9 of the studies using 3 ROM measures.

**Fig. 3:**
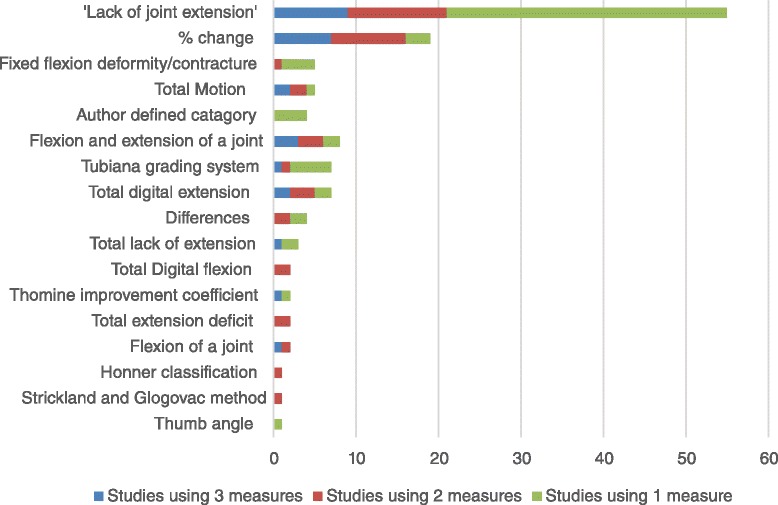
Variation of measures used by description (n = 17) with the number of measures used for each study

The 17 categories illustrate the varied use of ROM outcome measures used in Dupuytren’s disease publications (Fig. 3).

Fixed flexion deformity (FFD) or fixed flexion contracture (FFC) was reported in 5 studies [33, 34, 36, 66, 73]. The definition and calculation of FFD/FFC varied. Percentage change as a measure was used in 19 studies [17, 28, 30, 40–44, 50–52, 59, 66–68, 90, 91, 96, 99] with 3 reporting it as their sole outcome measure [52, 90, 91]. Four studies [20, 39, 45, 57, 58, 78, 89, 98, 99] presented data as differences or graphs with 2 authors using their chosen ROM outcome as their only measure [20, 39, 45, 57].

### Hyperextension

Hyperextension was included in 11 of the studies [11, 33, 50, 53, 66, 72, 75, 77, 80, 83, 89]. The description of how this was recorded and analysed varied between studies. Three studies reported hyperextension as a negative value [33, 50, 80] of which two [33, 80] subtracted and one added [50] hyperextension to the ROM calculation. Hyperextension measurement was included in results in four studies [11, 66, 77, 83] without prior description in the text or discussion on how hyperextension was analysed. Three studies [72, 75, 89] indicated that hyperextension was included in a composite ROM measure with only one [75] stating how hyperextension was calculated within the composite measure. One publication clearly described how hyperextension was recorded and analysed as a zero value [53].

### Grading systems & composite measures

Tubiana grading system assigns a score to the extension deficit of a digit by summing the total deficit angles of the MCP, PIP and distal interphalangeal (DIP) [101, 102]. Tubiana grading system was reported both pre and post operatively in 7 studies [19, 50, 55, 65, 72, 86, 93]. One study [50] used Tubiana in addition to another ROM measure, the remaining 6 studies [19, 55, 65, 72, 86, 93] used it as their sole measure.

Total active motion (TAM) is described by the American Society for Surgery of the Hand (ASSH) as the sum of active MCP, PIP and DIP arc of motion in degrees of an individual digit [6]. This calculation can then be compared to the TAM of the contralateral hand or the norm of 260 degrees. Five studies [23, 24, 48, 75, 89] reported using TAM methodology. Four [23, 24, 75, 89] of the 5 studies reporting Total Motion recorded active measurements supplemented with one or more ROM measures. One study used Total Motion as their only measure recording both active and passive motion [48]. Two of the 5 studies reporting TAM described their goniometer protocol [23, 48].

Other grading systems used were Thomine improvement coefficient [57, 89], Honner classification [58] and Strickland and Glogovac method [78].

## Discussion

It is reported that ROM is the most commonly reported outcome measure for Dupuytren’s disease [5] however it is clear from reviewing the literature that there is confusion regarding what to measure, method of measure and how to describe and report it. The study designs were retrospective (31 publications) or unclear (18 publications) for 49 of the 90 studies. Future publications should focus on clearly reporting prospective data using well-designed studies. This review was limited to publications in the English language and to studies that reported comparable ROM data to meet the aim of this review. The quality of the ROM method and the presentation of the results are now critiqued to identify robust ROM methods for future studies.

From reviewing the literature a number of concerns were identified regarding assessment and reporting of data that will now be discussed in turn.

- Use of terminology
- Measurement protocol
- Hyperextension reporting and analysis
- Inconsistencies in Total Active Motion
- Use of Tubiana Grading system
- Percentage change to demonstrate change
- Other methods

### Use of terminology

There were 8 ROM descriptors that appeared to describe the same measure (see Fig. 2). While this may appear to be semantics, lack of published protocols necessitates interpretation by the reader leading to potential ambiguity. For clarity authors should provide a clear protocol to support their chosen method.

From the literature reviewed it is suggested that the terminology currently in use is varied. From the thematic analysis 24 Dupuytren’s disease ROM categories were found. With the absence of measurement definition, 8 of these ROM categories appeared to be reporting the same measure (Lack of joint extension) as illustrated in Fig. 2.

There are inconsistencies in terminology within studies; the use of ‘contracture’ and ‘deformity’ was used interchangeably [76] and pre-operative ‘contracture’ and post-operative ‘joint extension’ [79]. Watt [95] clearly defined what they measured as ‘degree of extensor lag’ yet presented results as ‘joint contracture’. Another study [62] reported mean extension deficit at base line and the mean extension deficit gain post treatment. This lack of consistency causes uncertainly when comparing results between studies.

The term ‘fixed’ in FFD/FFC suggests an arthrodesed joint, unable to extend or flex from its fixed position. However, in the studies reviewed the term ‘FFD’/FFC has been identified in some of the studies to describe movable joints [33, 36, 44, 54, 66, 73, 98]. It is therefore anticipated that some authors referred to a joint with limited maximum extension rather than an arthrodesed joint. Only one study [34] described how FFC was calculated. This highlights the need for an agreed FFD/FFC definition to enable consistency. Until this is achieved future publications should clearly define what is meant by the term ‘fixed’. Authors need to be more aware of the choice of terminology to assist clearer communication of their findings.

### Measurement protocol

As goniometry has been previously acknowledged as the most frequent outcome measure [5] it can therefore be argued that a ROM protocol should be provided as standard in publications to aid transparency.

The position of the MCP joint can influence the maximum motion achieved at the PIP joint, as reported by Tonkin et al [103]. This also has the added benefit of minimizing the effect of dynamism as described by [104]. While passive motion is useful to record changes resulting from surgery, active motion may illustrate the functional gain. To demonstrate a comprehensive ROM of the Dupuytren’s hand we advocate the inclusion of active and passive digital flexion and extension of each joint, with the additional measure of active PIP extension with the MCP held in flexion.

The lack of clarity regarding if a joint was assessed actively or passively was unexpectedly high and illustrates the difficulty to accurately compare findings. This problem was also highlighted in a recent Cochrane review [4]. Both active and passive motion is important to establish severity in Dupuytren’s disease, improvement and potential for gain and those studies reporting both [77, 97] may provide deeper insight into the potential functional use of the hand.

The use of a goniometer in measuring ROM also highlights the need to define zero degrees. Witthaut [99] stated zero degrees on the goniometer as neutral whereas Lee [58] described full extension as 180 degrees. Usual practice defines full joint extension as zero degrees but either is acceptable when described.

Change in flexion may suggest functional impairment and was reported in only two studies [77, 96]. Weinzweig [96] reported active flexion pre and post-operatively concluding that flexion deteriorated. It is important that flexion is assessed and reported pre and post intervention to establish if extension has been achieved at the expense of flexion. Van Rijssen [90, 91] reported post-operative flexion deficit at 6 weeks with distance from palm to distal palmar crease but no pre-operative data, preventing analysis of change. It should not be presumed that flexion at baseline is unaffected.

### Hyperextension reporting and analysis

It is usual clinical practice to record hyperextension of a joint to document flexibility. Compensatory hyperextension of the MCP can be associated with an extension deficit at the PIP joint [89]. It may therefore be assumed that it is important to report any joint hyperextension as a potential indicator for biomechanical change.

How hyperextension is addressed in calculating ROM remains an issue. The difficulty arises when hyperextension of a joint is included in ROM analysis as it may produce misleading data. Including hyperextension when calculating MCP motion increases overall arc of motion [75]. Some authors considered hyperextension as a negative value in their calculation subtracting the hyperextension from the total digit extension deficit, producing a lower deficit [33, 80]. Conversely, adding hyperextension to post-operative gain at individual joints suggests an enhanced improvement [50]. Jerosch-Herold [53] recorded hyperextension, where present, and converted hyperextension to zero for TAE and TAF analysis purposes, thus avoiding problems highlighted above.

Where other authors expressed hyperextension denoting with a minus sign Denkler [31] used this annotation to express a lack of extension. In this case a pre-operative extension deficit is reported as a minus figure (e.g. -75). When a 45 degree improvement is recorded (therefore still short of full extension) this is illustrated as a smaller negative value (e.g. -30) differing to other studies. Universal agreement on the use of positive and minus values for extension and hyperextension recording and calculation is required to avoid confusion. An alternative solution is the use of ^H^ following the degrees measured, to record hyperextension e.g. 15^H^.

### Inconsistencies in total active motion

A disadvantage of TAM calculation means that change in any particular joint cannot be identified unless supplemented with additional information for transparency.

Only 2 studies [24, 48] reported calculating TAM as described by ASSH [6]. In contrast one study interpreted TAM as the arc of motion at each of the MCP and PIP joints [89] while another reported the use of TAM by combining the calculations of three digits (middle, ring and little) to provide an overall figure [23]. This combined TAM precludes comparison with other studies or analysis of the data for each joint and digit. The varied interpretations of TAM demonstrates inconsistencies further highlighting concerns. It is recommended that authors correctly use the originally described method of calculation in the future.

Transparent reporting of ROM was provided by Roush [75] in tables to clearly detail extension and flexion of each joint for each patient. However, hyperextension was used in the TAM calculation. An increase in hyperextension can be miss-interpreted as ROM gain [6] but may also conceal a decrease at another joint. While TAM has benefits in reporting the arc of motion it should always be supplemented with additional ROM measures for each joint as demonstrated by Roush [75]. As the PIP joint responds differently to the MCP joint in the disease and treatment process detailing the results of each joint individually will also enable further comparison.

Shaw [78] analysed ROM data using another composite measure [105] which sums the MCP and PIP (i.e. excludes the DIP). This has the advantage of only including the 2 joints primarily affected by Dupuytren’s disease, but ignores the secondary changes that may occur at the DIP joint. Providing the active flexion and extension measurements of all three joints is therefore the preferred option.

### Use of tubiana grading system

Tripoli [86] clearly reported using passive motion in calculating the digital Tubiana score. Referencing sources demonstrate implied application of a given methodology however, stating implicitly how it was followed reassures the reader. Hovius [50] reports calculating the Tubiana score by summing the MCP and PIP joints and does not state the inclusion of the DIP. Beaudreuil [19] calculates a Tubiana score taking the whole hand into consideration. This illustrates the variations in interpretation of the Tubiana methodology.

Tubiana calculation has been modified over time since its original conception [101, 102]. While this demonstrates development of the measure it also poses challenges for analysis with authors using a variety of published versions that may preclude comparison. Six of the 7 studies supported their selected Tubiana methodology with one of five references. There are inherent flaws when calculating Tubiana. As previously discussed including hyper extension in a composite calculation raises issues with analysis. It is unclear from the 7 studies [50, 55, 65, 72, 86, 93] how hyperextension was addressed. Additionally this is compounded with Tubiana [102] stating that the degree of hyperextension at the DIP joint is added to the total flexion deformity (i.e. extension deficit) of the other joints but is not supported with the example calculation.

A further difficulty with the Tubiana calculation is the assignment to a corresponding stage. This is apparent when the correct calculation falls between two ROM bands (e.g. 45 degrees at the top of stage 1 or bottom of stage 2 and 90 degrees in stage 2 or 3). The ROM bands for the Tubiana stages are clearly stated in 3 studies [55, 72, 86], however the authors do not state how they dealt with calculations that could be assigned to either stage. Improvement of ROM may occur within a band yet the measure lacks sensitivity to illustrate such.

The authors of this paper also argue that Tubiana calculations do not adequately describe at which joint a ROM deficit occurs. This is illustrated by Vigroux [93] when the figures report overall pre and post-operative Tubiana scores yet do not identify if this equates an improvement for the individual patient or trends in movement.

Tubiana was more often used as a method to grade severity of Dupuytren’s disease at baseline [12, 18, 41, 47, 67, 69, 87, 90, 91] with other ROM measures used pre and post intervention for comparative purposes. It is therefore suggested that Tubiana is best used to identify baseline disease severity or to stratify for analysis rather than as an outcome measure.

### Percentage change

Percentage change was used in 3 [52, 90, 91] studies as their sole measure. Percentage change is arguably of little value unless baseline data is presented for context. For example a 90 % improvement in extension deficit may be illustrated by one patient improving from 20 degrees extension deficit at baseline to 2 degrees while a second patient improves from 80 to 8 degrees. The second example gained the larger improvement (72 degrees), started further from zero degrees (desired end point) and although the results share common metrics their comparison lacks context. It is therefore suggested that the use of percentage change is not used as the sole outcome measure.

### Other ROM analysis

Four studies presented results as the differences for their chosen outcome measure [39, 45, 98, 99]. In presenting differences, rather than the actual measurements for at least one of the data sets, means that the pre or post ROM cannot be established for comparison proposes. One study presented data differences in graphs [39] therefore preventing accurate data extraction. We would recommend that future studies include at least one tabled data set if reporting differences as results.

Four studies presented results using 3 other grading systems Thomine improvement coefficient [57, 89], Honner classification [58] and Strickland and Glogovac method [78]. Only 1 study evaluated thumb angles using distance measured in centimetres [20] (see Fig. 3).

Using an outcome measure not commonly used by other studies presents difficulty when comparing results for meta-analysis.

## Conclusion

Based on our findings we recommend that ROM measuring and recording for Dupuytren’s disease requires consistency to enable comparability of results in future research. All studies should include active and passive digital flexion and extension of each joint, with the additional measure of active PIP extension with the MCP held in flexion. The following points should also be considered as recommended in Table 3.

**Table 3:** Recommendations to improve ROM measuring and reporting robustness in practice

Definition of terms
1. Agreement on terminology to report ‘lack of extension’ at a joint.
2. Agreed Fixed Flexion Deformity definition to enable consistency.
Protocol statement
3. Adherence to an agreed published ROM protocol to aid transparency
4. Zero or 180 degrees should be considered equivalent when describing full extension for reporting and analysis if previously described.
5. Universal agreement is required on the use of positive or negative values in hyperextension measurement for reporting and analysis.
Outcome reporting
6. Clear and consistent use of reporting active / passive ROM is required for comparison of data.
7. Flexion should be assessed and reported pre and post intervention to establish if extension was achieved at the expense of flexion.
8. When reporting composite measures (e.g. TAM, Tubiana) additional ROM measures for each joint should be recorded to identify where the change occurred.
9. Further clarity is required for Tubiana band definitions to avoid overlapping.
10. Percentage change should not to be used as the sole outcome measure without baseline data.
11. Outcomes presented as differences must be supported with at least one tabled data set for accurate reporting.
12. ROM presented in graphs must be supported by data for accurate reporting.

We also encourage the use of standard terminology in assessing Dupuytren’s disease to further define the ROM assessment. It is suggested if the issues1-5 raised in Table 3 are addressed by professional bodies (Definition of term and Protocol Statement) it will assist in the development of consistent procedures. Researchers and clinicians can also play a part in improving the quality of Dupuytren’s disease evidence by addressing points 6-12 highlighted in Table 3. Together by addressing these issues ROM outcomes in research can be improved which will provide better quality evidence in future publications [3, 8, 9].

## Additional file

Additional file 1:
**PRISMA Checklist: PRISMA Checklist of items to include when reporting a systematic review or meta-analysis.** (DOC 69 kb)
